# Prenatal Ultrasonographic Markers of Macrossomia and C-Peptide in Gestational Diabetes Mellitus: A Prospective Cohort Study

**DOI:** 10.3390/diagnostics15161989

**Published:** 2025-08-08

**Authors:** Roberto Noya Galluzzo, Karine Souza Da Correggio, Aldo von Wangenheim, Gustavo Yano Callado, Heron Werner, Edward Araujo Júnior, Pedro Teixeira Castro, Glória Calagna, Alexandre Sherlley Casimiro Onofre

**Affiliations:** 1 Division of Tocogynecology, University Hospital Polydoro Ernani of São Thiago, Federal University of Santa Catarina (UFSC), Florianópolis 88036-800, Brazil; rngalluzzo@gmail.com (R.N.G.); kdacoregio@gmail.com (K.S.D.C.); 2 Brazilian Institute for Digital Convergence, Federal University of Santa Catarina (UFSC), Florianópolis 88040-900, Brazil; aldo.vw@ufsc.br; 3 Discipline of Woman Health, Albert Einstein Israelite College of Health Sciences (FICSAE), Albert Einstein Israelite Hospital, São Paulo 05653-000, Brazil; gycallado@gmail.com; 4 Department of Fetal Medicine, Biodesign Laboratory DASA/PUC, Rio de Janeiro 22451-900, Brazil; heron.werner@gmail.com (H.W.); pedrotcastro@gmail.com (P.T.C.); 5 Department of Obstetrics, Paulista School of Medicine, Federal University of São Paulo (EPM-UNIFESP), São Paulo 04023-062, Brazil; araujojred@terra.com.br; 6 Discipline of Woman Health, Municipal University of São Caetano do Sul (USCS), São Caetano do Sul 09521-160, Brazil; 7 Villa Sofia Cervello Hospital, University of Palermo, 90100 Palermo, Italy; 8 Department of Clinical Analyses, Federal University of Santa Catarina (UFSC), Florianópolis 88036-800, Brazil; alexandre.onofre@gmail.com

**Keywords:** gestational diabetes mellitus, prenatal ultrasound, neonatal hyperinsulinemia, C-peptide

## Abstract

**Objective:** To investigate the association between prenatal ultrasonographic markers of macrossomia and C-peptide, a neonatal hyperinsulinemia marker, in pregnancies complicated by gestational diabetes mellitus (GDM), with a focus on fetal adipose tissue thickness, liver length, and interventricular septal thickness. **Methods**: This prospective cohort study included 223 pregnant women followed from 28 to 36 weeks of gestation in two referral centers in Brazil. The GDM group and matched controls underwent serial ultrasound assessments of fetal biometry, including thigh, abdominal, and subscapular skinfolds, fetal liver length, and interventricular septum thickness. Neonatal hyperinsulinemia was assessed using umbilical cord C-peptide levels. Statistical analyses included *t*-tests, chi-square tests, correlation analyses, and multivariate logistic regression. **Results**: Fetuses of mothers with GDM exhibited significantly greater abdominal [t(221) = −3.999, *p* < 0.01] and subscapular [t(221) = −2.502, *p* = 0.02] skinfolds, liver length [t(221) = −3.785, *p* < 0.01], and interventricular septum [t(221) = −4.781, *p* < 0.01] thickness. However, umbilical cord C-peptide levels did not differ significantly between groups [t(189) = −1.724, *p* = 0.09]. Only weak correlations were found between fetal ultrasound markers and C-peptide levels. Among all parameters, subcutaneous tissue thickness showed the highest (ρ = 0.30), though still limited, predictive value. **Conclusions:** Fetuses of mothers with GDM demonstrated increased measures of liver length, subscapular adiposity, and interventricular septal thickness compared to controls. However, these prenatal biometric markers showed weak correlations with neonatal C-peptide levels.

## 1. Introduction

Gestational Diabetes Mellitus (GDM) presents an increased risk of maternal and fetal mortality and morbidity. The fetal environment of hyperglycemia and hyperinsulinemia, induced by maternal hyperglycemia [1], exposes the fetus to an elevated risk of postnatal neonatal hypoglycemia in the first hours after birth, with the possibility of brain injury and subsequent neurodevelopmental impairment [2]. The identification of fetuses at risk of neonatal hypoglycemia may reduce the risk of this condition and its consequences [3].

Among markers of neonatal hyperinsulinemia, human connecting peptide (C-peptide) has been evaluated as an independent risk factor for neonatal hypoglycemia. The cleavage of proinsulin produces the insulin molecule and C-peptide in the Golgi complex of pancreatic beta cells. Insulin and C-peptide are stored in equal proportions, and both molecules are released in equal amounts into the portal circulation after a glycemic stimulus. However, the difference in molecular stability (insulin has a 3 min half-life, while C-peptide has a 30 min half-life) makes C-peptide a reliable marker of pancreatic beta-cell function [4].

In perinatal medicine, C-peptide has been suggested as a potential marker for neonatal metabolic outcomes. In GDM, umbilical cord levels of C-peptide are associated with fetal macrosomia [5]. In type 1 diabetes pregnancies, umbilical cord C-peptide levels are an independent risk factor for neonatal hypoglycemia [6]. Fetal subcutaneous tissue thickness (SCTT), measured at the abdominal wall, subscapular region, and as a ratio to femur length, is a useful marker of fetal growth and maternal glycemic control. Studies have shown higher SCTT in fetuses of mothers with GDM, with values decreasing after treatment. Chen et al. [7] proposed reference ranges for SCTT between 21 and 36 weeks in healthy pregnancies, supporting its use in predicting macrosomia and GDM-related overgrowth.

In pregnancies complicated by GDM, ultrasound presents moderate accuracy in identifying fetuses with macrosomia, with sensitivity of 71% and specificity of 88%. Estimated fetal weight has sensitivity and specificity of 76% and 83%, respectively, while abdominal circumference has sensitivity of 84.8% and specificity of 73% [8]. Among ultrasound markers of macrosomia, the SCTT-to-femur length ratio has shown greater accuracy in identifying large-for-gestational-age (LGA) fetuses than abdominal circumference or estimated fetal weight [9]. It is simple, gestational-age-independent, and less prone to false positives and negatives, although it may misclassify cases involving low birth weight, obesity, or long neonates with normal fat.

Hypertrophy of the interventricular septum is another marker of fetal hyperinsulinemia. In diabetic pregnancies, insulin acts on myocardial receptors, leading to septal thickening regardless of fetal weight. Although cellular disorganization is absent, hydropic changes may occur. Cardiac involvement depends on maternal glycemic control, emphasizing the need for close metabolic monitoring [10].

Fetal liver length is often increased in pregnancies complicated by type 1 or type 2 diabetes, independent of maternal size. This disproportionate growth, likely linked to hyperinsulinemia, may be evident as early as 18 weeks and tends to regress with improved glycemic control. It represents a specific and early marker of fetal metabolic adaptation in diabetic pregnancies [11,12].

The primary objective of this study was to assess the association between prenatal ultrasonographic markers of macrosomia and neonatal hyperinsulinemia in pregnancies complicated by suspected GDM. Specifically, it aimed to determine whether fetal adipose tissue measurements—such as thigh, abdominal, and subscapular skinfold thickness—are associated with biochemical evidence of neonatal hyperinsulinemia. In addition, the study evaluated fetal liver length and interventricular septal thickness, correlating these morphometric parameters with postnatal markers of fetal hyperinsulinemia.

## 2. Methods

### 2.1. Study Design and Setting

This was a prospective cohort study conducted from March 2022 to March 2024, aiming to evaluate the association between prenatal ultrasonographic markers and markers of neonatal hyperinsulinemia (C-peptide). Participants were recruited and followed at the high-risk prenatal outpatient clinics of Carmela Dutra Maternity and the University Hospital Polydoro Ernani of São Thiago of the Federal University of Santa Catarina (UFSC), Brazil. The control group was recruited from the routine-risk prenatal clinic of the same university hospital.

### 2.2. Participant Selection

Pregnant women aged 18 to 45 years, residing in the metropolitan region of Florianópolis, Brazil, and diagnosed with GDM were eligible for inclusion if they received prenatal care at high-risk clinics and signed the informed consent form. The control group consisted of an equal number of low-risk pregnant individuals without known maternal or fetal risk factors. Controls were matched based on similar maternal characteristics. Exclusion criteria included multiple gestations or fetal structural anomalies. A census approach was adopted, enrolling all eligible patients with GDM within the study period.

GDM was diagnosed according to the Brazilian Ministry of Health protocol, either through a 75 g oral glucose tolerance test (OGTT) or based on a fasting plasma glucose level ≥92 mg/dL on at least two separate measurements. In cases where the OGTT was not performed (*n* = 75), clinical diagnosis was established based on elevated fasting or random glucose values documented by the prenatal care provider.

### 2.3. Data Collection

Eligible participants were invited to join the study during routine prenatal visits. After signing the informed consent form, clinical and demographic data were collected using a standardized instrument and their prenatal cards were labeled to ensure identification at the time of delivery for continued data collection. Data collection occurred at three distinct time points. The first encounter, at enrollment, involved gathering baseline demographic and clinical information, including age, ethnicity, parity, gestational age (GA), GA at GDM diagnosis, comorbidities, lifestyle habits (such as physical activity, tobacco, alcohol or drug use, and dietary adjustments for diabetes), pre-pregnancy weight, body mass index (BMI), current medication use, and family history of diabetes.

**Figure 1:**
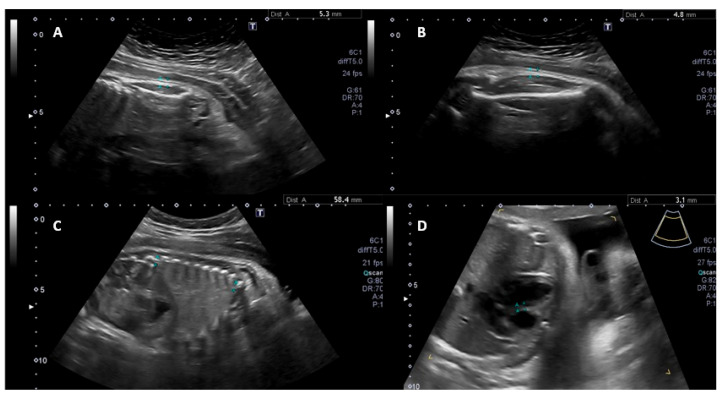
(**A**) Fetal scapular subcutaneous measurement; (**B**) fetal thigh subcutaneous measurement; (**C**) fetal liver length measurement; and (**D**) fetal interventricular septum measurement.

The second and third encounters, conducted between 28–32 and 32–36 weeks of gestation, included detailed ultrasonographic assessments. These comprised measurements of fetal skinfold thickness, liver size and echotexture (Figure 1), abdominal-to-head circumference ratio (AC/HC), peak systolic velocity (PSV) of the middle cerebral artery (MCA) Doppler, ductus venosus flow Doppler, and interventricular septum thickness, along with routine biometric and Doppler parameters such as amniotic fluid index and umbilical artery Doppler. At these visits, data were also collected on the participant’s glycemic profile and insulin use. All fetal ultrasound assessments were performed by two experienced maternal-fetal medicine specialists, each with more than five years of experience. Both sonographers adhered to standardized protocols, and regular joint calibration sessions were held to reduce interobserver variability.

Following delivery, umbilical cord blood was collected and analyzed in the respective institutional laboratories to assess C-peptide levels, complete blood count, and bilirubin levels. Additional perinatal data included GA at delivery, labor induction, delivery mode, indication for cesarean section, and intrapartum complications such as shoulder dystocia and postpartum hemorrhage. From the neonatal records, data were extracted on the newborn’s sex, birth weight and length, Apgar scores, episodes of neonatal hypoglycemia, respiratory distress, need for neonatal intensive care unit (NICU) admission, intubation, brachial plexus injury, clavicle fracture, and neonatal death.

### 2.4. Ethical Considerations

The study was approved by the Human Research Ethics Committee of UFSC (CAAE 49990921.2.1001.0121) and conducted in accordance with national ethical guidelines. All participants provided written informed consent. Confidentiality, privacy, and autonomy were ensured throughout the study, and the anticipated risk was minimal. Interviews were conducted in private settings to protect sensitive information.

### 2.5. Data Analysis

Data were entered into an Excel^®^ 2010 spreadsheet (Microsoft Corp., Redmond, WA, USA) and analyzed using IBM SPSS Statistics, version 21 (IBM Corp., Armonk, NY, USA). Descriptive statistics were used to summarize the variables, with means, standard deviations, frequencies, and percentages reported according to variable type.

To address non-normal distributions and increase reliability, bootstrap resampling (500 iterations; 95% bias-corrected and accelerated confidence intervals) was employed. Associations were reported as odds ratios (ORs) with corresponding 95% confidence intervals (CIs) and *p*-values. A significance level of *p* < 0.05 was adopted for all analyses.

Pearson’s chi-square test was used for comparisons of categorical variables between groups, and Student’s *t*-test was applied for comparisons of continuous variables. Univariate binary logistic regression models were first used to assess the crude association between each predictor and the primary outcome, defined as C-peptide levels above the 75th percentile. Variables with *p* < 0.20 in univariate analysis were included in the multivariate logistic regression to identify independent predictors while controlling for confounding factors.

## 3. Results

A total of 223 participants were included in the study, with 95 (42.6%) allocated to the control group and 128 (57.4%) to the GDM group. Participant age ranged from 18 to 43 years, with a mean of 28.2 ± 5.9 years. The mean BMI was 28.0 ± 6.3 kg/m^2^, and the most frequent BMI category was overweight, representing 30.9% of the sample. The majority of participants identified as white (74.0%), reported no alcohol consumption (95.1%), no illicit drug use (98.7%), no smoking (94.6%), and no regular physical activity (80.7%) (Table 1). These variables were included to characterize the population and to assess potential confounders.

Mean weight gain during pregnancy was 12.0 ± 7.4 kg, and weight gain was classified as inadequate in 71.7% of participants. The mean gestational age at delivery was 276.4 ± 9.6 days. Most participants were primigravidas, had no previous cesarean sections, and did not receive prenatal care at the university hospital. A family history of type 2 diabetes was uncommon.

Among the 128 women with GDM, 41.4% received the diagnosis during the second trimester. Insulin therapy was prescribed in 27.3% of cases, with 54.5% of these starting treatment in the third trimester. Notably, 58.6% of women did not undergo the OGTT, and over half (55.2%) were not following an appropriate dietary plan (Table 2).

Analysis of fetal morphometric markers revealed significant group differences. Fetuses of GDM mothers presented with significantly greater abdominal skinfold thickness [t(221) = −3.999, *p* < 0.01], liver length [t(221) = −3.785, *p* < 0.01], subscapular skinfold thickness [t(221) = −2.502, *p* = 0.02], and interventricular septum thickness [t(221) = −4.781, *p* < 0.01] compared to those in the control group. Although thigh skinfold thickness was also higher in the GDM group, this difference did not reach statistical significance (*p* = 0.07) (Table 3).

When categorizing fetal biometric parameters as normal or altered, a significantly higher frequency of abnormalities was observed in the GDM group for the thigh-to-femur skinfold ratio (*p* = 0.009), liver length (*p* = 0.02), subscapular skinfold thickness (*p* = 0.03), and interventricular septum thickness (*p* = 0.008). In contrast, no significant difference was observed in the proportion of abnormal abdominal skinfold measurements between the groups (*p* = 0.29) (Table 4).

Regarding neonatal C-peptide levels, the mean value was 1.1 ± 0.6 ng/dL, with no statistically significant difference between the GDM and control groups [t(189) = −1.724, *p* = 0.09]. Additionally, 60.2% of neonates had C-peptide levels below the 75th percentile, with no significant group differences in this distribution [χ^2^(1) = 2.097, *p* = 0.15]. Spearman correlation analysis revealed weak but positive associations between C-peptide levels and thigh skinfold thickness (ρ = 0.30), abdominal skinfold thickness (ρ = 0.27), liver length (ρ = 0.23), subscapular skinfold thickness (ρ = 0.16), and interventricular septum thickness (ρ = 0.10) (Table 5).

## 4. Discussions

Pregnancy is known to be a condition of physiological insulin resistance due to the unique fetal metabolism. The increased incidence of overweight and obesity in recent decades has raised the incidence of GDM, reaching 9% of pregnancies in the USA [13]. Excessive maternal adiposity and a hyperglycemic state predispose offspring to an elevated risk of metabolic conditions later in life [14,15]. Even in the absence of maternal diabetes, a hyperglycemic state during pregnancy increases the risk of adverse metabolic outcomes in offspring [16].

C-peptide has been described as a marker for studying neonates of diabetic mothers for more than 50 years [17]. As a marker of hyperinsulinemia, umbilical cord C-peptide concentrations correlate with maternal C-peptide levels during pregnancy as well [16]. Despite the fact that maternal C-peptide might be related to neonatal hyperinsulinemia, in this study the ultrasound markers of fetal macrosomia demonstrated poor statistical correlation with neonatal C-peptide levels.

The findings of this prospective cohort study indicate that specific prenatal ultrasonographic markers—namely fetal liver length, subscapular skinfold thickness, and interventricular septal thickness—were significantly increased in fetuses of mothers with GDM compared to controls. Although thigh skinfold thickness was also elevated in the GDM group, it did not reach statistical significance. Importantly, there was no significant difference in neonatal C-peptide levels between the groups, and only weak correlations were observed between fetal morphometric parameters and postnatal biochemical evidence of hyperinsulinemia. These results suggest that, although certain fetal morphologic changes are more frequent in GDM pregnancies, their predictive value for neonatal hyperinsulinemia remains limited when considered in isolation.

Previous studies have consistently demonstrated that fetal subcutaneous tissue thickness, particularly at the abdominal wall and subscapular regions, is strongly associated with neonatal hyperinsulinemia and adverse metabolic outcomes in pregnancies complicated by GDM—even when glycemic control is adequate and macrosomia is absent [18,19,20]. Correlations between SCTT and umbilical cord blood insulin, C-peptide, and the Homeostasis Model Assessment of Insulin Resistance (HOMA-IR) have been reported across diverse populations, suggesting a robust physiological relationship between fetal fat accretion and in utero hyperinsulinemia [20,21,22,23,24]. In contrast, the present study found only weak correlations between these morphometric parameters and neonatal C-peptide levels. This discrepancy may be due to differences in the timing of ultrasound assessments, sample size, or the single-point measurement of C-peptide at delivery, which may not fully capture fetal insulin dynamics across gestation. Nonetheless, our findings reinforce the idea that increased subcutaneous fat is common in fetuses exposed to GDM, but its isolated measurement may not reliably predict neonatal hyperinsulinemia.

Fetal interventricular septal (IVS) thickness has been widely studied as a marker of cardiac remodeling in diabetic pregnancies. It is well established that IVS hypertrophy reflects fetal exposure to hyperinsulinemia, particularly in cases of poor maternal glycemic control [25,26,27]. Although IVS is consistently elevated in GDM, its clinical relevance as a predictor of neonatal cardiac dysfunction or long-term metabolic risk remains uncertain [28]. Several studies have shown that IVS thickening is not typically accompanied by overt cardiac dysfunction at birth, and functional impairments may be subclinical or transient [29,30]. In line with these findings, our study found significantly increased IVS thickness in the GDM group but only a weak correlation with neonatal C-peptide levels, suggesting that IVS may reflect fetal exposure to hyperinsulinemia without offering direct predictive value for metabolic outcomes.

Fetal liver length is another parameter commonly altered in GDM pregnancies. Prior studies have linked liver enlargement with maternal glycemic status and fetal macrosomia, and some have shown associations with umbilical cord blood metabolic markers [31,32,33]. However, liver length remains a nonspecific marker with limited predictive power for neonatal hyperinsulinemia. This limitation is due in part to measurement variability, lack of established reference values, and inconsistent correlations with biochemical endpoints [11,34]. In our study, fetal liver length was significantly greater in the GDM group, consistent with the literature, but the correlation with C-peptide was again weak. These results underscore the need to interpret liver size alongside more metabolically informative parameters, such as SCTT, when evaluating fetal metabolic risk in GDM [35].

Although multiple morphometric parameters were evaluated in this study, including subcutaneous tissue thickness, liver length, and interventricular septal thickness, current evidence does not support the combined use of these markers as a superior strategy for predicting neonatal metabolic outcomes. Fetal subcutaneous tissue thickness remains the most robust and consistently validated ultrasonographic marker for identifying neonatal hyperinsulinemia and insulin resistance in pregnancies complicated by GDM, particularly when abdominal circumference is increased [18,35]. In contrast, fetal liver length, while associated with maternal glycemic control and fetal macrosomia, lacks predictive accuracy for neonatal hyperinsulinemia when SCTT is accounted for [33,36,37]. Similarly, interventricular septal thickness, although a reliable indicator of fetal cardiac remodeling, does not independently predict adverse metabolic outcomes or insulin resistance in the neonate [25,27,38]. To date, no high-quality evidence demonstrates that combining these three markers provides incremental predictive value beyond SCTT alone. Therefore, SCTT remains the preferred ultrasonographic parameter for assessing fetal metabolic alterations in GDM pregnancies.

From a clinical implementation standpoint, SCTT is the most feasible and reproducible ultrasonographic marker for routine use, even in low-resource or non-tertiary care settings. It can be reliably measured during standard second- or third-trimester scans with conventional equipment and demonstrates good intra- and interobserver reproducibility, particularly at the abdominal level [33,35]. Fetal liver length is also measurable with standard ultrasound but is more susceptible to technical limitations such as fetal position, maternal habitus, and operator experience. Although protocols exist, liver measurement is less standardized than SCTT, resulting in greater variability between observers [33,36,37]. In contrast, interventricular septal thickness (IVS) assessment requires more advanced expertise in fetal echocardiography and is less reproducible outside specialized centers. While technically feasible, its utility in general practice is limited by variability in technique and interpretation [25,30,38]. These practical considerations suggest that, among the evaluated markers, SCTT is the most accessible and clinically applicable for broad implementation in the prenatal evaluation of pregnancies affected by gestational diabetes.

This study has several limitations that should be acknowledged. First, the lack of universal OGTT administration in the GDM group may have led to underdiagnosis or misclassification of glycemic control, introducing potential bias in the characterization of maternal metabolic status. Additionally, dietary adherence and insulin use were self-reported, limiting the precision of glycemic control assessment. Second, while C-peptide is a validated marker of fetal insulin production, the single measurement at birth may not fully reflect in utero glycemic dynamics across gestation. Third, although this study used a robust and prospective design, the modest sample size, particularly when stratifying abnormalities by marker, may have limited the power to detect associations. Finally, the study was conducted at tertiary referral centers, which may limit the generalizability of the findings to broader obstetric populations.

While multiple biometric changes were observed in GDM-exposed fetuses, their individual predictive power for neonatal hyperinsulinemia—assessed by umbilical cord C-peptide—was limited. Among the evaluated parameters, subcutaneous tissue thickness, particularly in the abdominal and subscapular regions, showed the most consistent association and remains the most promising for clinical application.

## 5. Conclusions

In conclusion, the fetuses of mothers with gestational diabetes demonstrated increased liver length, subscapular adiposity, and interventricular septal thickness compared to controls. However, these prenatal ultrasonographic findings showed weak correlations with neonatal C-peptide levels. While these markers may serve as adjuncts in assessing the fetal metabolic effects of maternal diabetes, their isolated use as predictors of neonatal hyperinsulinemia appears limited. Further studies with larger populations and longitudinal metabolic profiling are needed to validate these findings and explore their potential clinical applications.

## Figures and Tables

**Table 1:** Baseline characteristics of study participants, stratified by group.

Variables	Group	
	Control (*n* = 95)	GDM (*n* = 128)
Age (years)	27.0 ± 5.8	29.0 ± 5.8
BMI (Kg/m^2^)	26.1 ± 5.2	29.4 ± 6.7
BMI Categories (%)		
Underweight	3 (3.2)	1 (0.8)
Normal weight	38 (40.0)	36 (28.1)
Overweight	31 (32.6)	38 (29.7)
Obesity class I	16 (16.8)	25 (19.5)
Obesity class II	6 (6.3)	18 (14.1)
Obesity class III	1 (1.1)	10 (7.8)
Race (%)		
White	71 (74.7)	94 (73.4)
Non-White	24 (25.3)	34 (26.6)
Alcohol use (%)		
No	87 (91.6)	125 (97.7)
Yes	8 (8.4)	3 (2.3)
Use of illicit drugs (%)		
No	95 (100.0)	125 (97.7)
Yes	0 (0.0)	3 (2.3)
Smoking (%)		
No	86 (90.5)	125 (97.7)
Yes	9 (9.5)	3 (2.3)
Physical activity (%)		
No	84 (88.4)	96 (75.0)
Yes	11 (11.6)	32 (25.0)

GDM = gestational diabetes mellitus; BMI = body mass index.

**Table 2:** Gestational age at diagnosis and dietary habits.

Variables	*n* (%)
Gestational age at diagnosis	
1st trimester	50 (39.1)
2nd trimester	53 (41.4)
3rd trimester	25 (19.5)
Gestational age at initiation of insulin therapy ^*^	
2nd trimester	15 (45.5)
3rd trimester	18 (54.5)
OGTT	
No	75 (58.6)
Yes	53 (41.4)
Adequate diet *	
No	64 (55.2)
Yes	52 (44.8)

* Variable with missing data. OGTT = oral glucose tolerance test.

**Table 3:** Comparative analysis of fetal parameters between gestational diabetes mellitus group and control group.

Variables	Mean ± SD	Mean Difference	*p*-Value *	95% CI of the Mean Difference
Thigh skinfold				
Control group	5.5 ± 1.2	−0.34	0.07	−0.70; 0.02
GDM group	5.8 ± 1.4			
Abdominal skinfold				
Control group	6.2 ± 1.3	−0.70	<0.01	−1.05; −0.36
GDM group	6.9 ± 1.3			
Liver length				
Control group	49.5 ± 7.2	−3.79	<0.01	−5.70; −1.98
GDM group	53.3 ± 7.5			
Subscapular skinfold				
Control group	6.6 ± 1.8	−0.61	0.02	−1.11; −0.13
GDM group	7.2 ± 1.8			
Interventricular septum				
Control group	5.5 ± 1.0	−0.65	<0.01	−0.92; −0.37
GDM group	6.1 ± 1.0			

* Independent samples Student’s *t*-test. GDM—gestational diabetes mellitus; SD—standard deviation; CI—confidence interval.

**Table 4:** Comparison of fetal measurements between gestational diabetes mellitus group and control group.

Variables	Group		*p*-Value *
	Control (*n* = 95)	GDM (*n* = 128)	
Thigh-to-femur skinfold ratio			0.009
Normal	64 (50.0)	64 (50.0)	
Altered	31 (32.6)	64 (67.4)	
Abdominal skinfold			0.29
Normal	92 (43.4)	120 (56.6)	
Altered	3 (27.3)	8 (72.7)	
Liver length			0.02
Normal	91 (45.3)	110 (54.7)	
Altered	4 (18.2)	18 (81.8)	
Subscapular skinfold			0.03
Normal	83 (46.1)	97 (53.9)	
Altered	12 (27.9)	31 (72.1)	
Interventricular septum			0.008
Normal	20 (64.5)	11 (35.5)	
Altered	75 (39.1)	117 (60.9)	

* Pearson’s Chi-square test.

**Table 5:** Spearman correlation coefficients between C-Peptide and fetal biometric measurements.

C-Peptide	Thigh Skinfold	Abdominal Skinfold	Liver Length	Subscapular Skinfold	Interventricular Septum
C-Peptide	–				
Thigh Skinfold	0.30	–			
Abdominal Skinfold	0.27	0.41	–		
Liver Length	0.23	0.19	0.24	–	
Subscapular Skinfold	0.16	0.34	0.39	0.22	–
Interventricular Septum	0.10	0.31	0.44	0.19	0.32

Values are presented as Spearman’s ρ correlation coefficients.

## Data Availability

The data presented in this study are available on request from the corresponding author.
